# Dead Chicken Identification Method Based on a Spatial-Temporal Graph Convolution Network

**DOI:** 10.3390/ani16030368

**Published:** 2026-01-23

**Authors:** Jikang Yang, Chuang Ma, Haikun Zheng, Zhenlong Wu, Xiaohuan Chao, Cheng Fang, Boyi Xiao

**Affiliations:** 1 Guangdong Laboratory for Lingnan Modern Agriculture, College of Engineering, South China Agricultural University, Guangzhou 510642, China; yjkscau@stu.scau.edu.cn (J.Y.); macscau@gmail.com (C.M.); xhchao@scau.edu.cn (X.C.); 2 School of Mechanical and Energy Engineering, Guangdong Ocean University, Yangjiang 529500, China; haikun0619@gmail.com; 3 Faculty of Bioscience Engineering, Katholieke Universiteit Leuven (KU LEUVEN), Kasteelpark Arenberg 30, 3001 Leuven, Belgium; zhenlong.wu@kuleuven.be; 4 College of Animal Science, South China Agricultural University, Guangzhou 510642, China; 5 State Key Laboratory of Livestock and Poultry Breeding, South China Agricultural University, Guangzhou 510642, China

**Keywords:** caged dead chicken, spatial-temporal graph convolution network, pose estimation, multimodal fusion

## Abstract

In intensive cage rearing systems, accurate dead hen detection remains difficult due to complex environments, severe occlusion, and the high visual similarity between dead hens and live hens in a prone posture. To address these issues, this study proposes a dead hen identification method based on a Spatial-Temporal Graph Convolutional Network (STGCN). Unlike conventional static image-based approaches, the proposed method introduces temporal information to enable dynamic spatial-temporal modeling of hen health states. First, a multimodal fusion algorithm is applied to visible light and thermal infrared images to strengthen multimodal feature representation. Then, an improved YOLOv7-Pose algorithm is used to extract the skeletal keypoints of individual hens, and the ByteTrack algorithm is employed for multi-object tracking. Based on these results, spatial-temporal graph-structured data of hens are constructed by integrating spatial and temporal dimensions. Finally, a spatial-temporal graph convolution model is used to identify dead hens by learning spatial-temporal dependency features from skeleton sequences. Experimental results show that the improved YOLOv7-Pose model achieves an average precision (AP) of 92.8% in keypoint detection. Based on the constructed spatial-temporal graph data, the dead hen identification model reaches an overall classification accuracy of 99.0%, with an accuracy of 98.9% for the dead hen category. These results demonstrate that the proposed method effectively reduces interference caused by feeder occlusion and ambiguous visual features. By using dynamic spatial-temporal information, the method substantially improves robustness and accuracy of dead hen detection in complex cage rearing environments, providing a new technical route for intelligent monitoring of poultry health status.

## Simple Summary

This paper introduces a practical vision system for early, reliable dead hen detection in caged layer houses. Using synchronized visible and thermal infrared cameras with geometric alignment, it extracts hen keypoint trajectories via pose estimation and tracking, then applies a spatiotemporal graph convolutional network for video level decisions. The method is designed to reduce false alarms caused by occlusion and by confusing “resting” with “dead.” Experiments show strong accuracy across challenging barn conditions, supporting faster inspection, timely removal, and improved welfare management in large-scale farms.

## 1. Introduction

Chicken production is of global importance because chickens represent a major source of animal protein for human consumption [1,2]. With continuous development, the chicken farming industry in China has gradually shifted toward large-scale and intensive production systems. Under such intensive rearing conditions, accurate and continuous monitoring of chicken health status becomes a labor intensive and technically demanding task [3,4]. Among various health management tasks in caged systems, dead hen detection is one of the most critical. Timely identification and removal of dead hens is essential for maintaining environmental hygiene and reducing disease transmission risks in poultry production [5,6].

Computer vision techniques have been widely applied to chicken health monitoring, and most existing studies rely on image data combined with object detection models [7]. Commonly used image modalities include visible light and thermal infrared images, with visible light images being the most frequently adopted. In the early stage of machine learning applications, classical machine learning algorithms and OpenCV-based image processing methods were employed for dead hen detection [8]. Zhu et al. (2009) [9] proposed an automatic dead hen detection method based on a support vector machine, in which comb-related parameters were used as feature vectors for classification. With the development of deep learning, object detection networks have become the dominant solution. Hao et al. (2022) [10] developed a dead broiler inspection system based on the YOLOv3 network that can be deployed on autonomous inspection robots, achieving an average precision of 98.6%. Cao et al. (2024) [11] proposed an improved YOLOv5-based method for automatic identification of broiler mortality in caged environments by modifying the network structure and applying model pruning to reduce parameter size and computational cost. Tong et al. (2023) [12] introduced a modified YOLO-based framework for chicken health status detection in which the improved YOLOv5l model achieved a maximum average precision of 94.9% with a detection speed of 34 frames per second (FPS). Yang et al. (2024) [13] proposed a lightweight and accurate dead hen detection method based on an improved YOLOv7 model, achieving a detection accuracy of 95.7% through optimization of the backbone network, loss function, and non-maximum suppression strategy combined with knowledge distillation.

Visible light images provide rich color information, whereas thermal infrared images describe thermal radiation characteristics through the temperature distribution [14]. Near-infrared images exhibit stronger robustness to illumination variation. Each image modality provides different feature information for dead hen detection. To overcome the limitations of single modality feature representation, several studies have introduced multimodal image fusion strategies to improve detection performance. By exploiting the complementary characteristics of different modalities, detection accuracy and robustness can be improved. Luo et al. (2023) [15] proposed a multimodal dead hen detection method based on the fusion of thermal infrared, near-infrared, and depth images, and compared detection performance across different modality combinations. Their results indicated that near-infrared depth and near-infrared thermal infrared dual modality images achieved better overall performance when considering both accuracy and speed. Sun et al. (2025) [16] proposed a multimodal detection method for diseased caged hens by integrating behavioral and thermal features through instance segmentation, achieving average precision values of 99.3% and 96.1% for head and leg regions in infrared images, respectively. Jiang et al. (2025) [17] developed a dead broiler detection method using infrared visible image fusion and computer vision techniques, achieving a detection success rate of 96.67% in commercial broiler farms with a single frame processing time below 0.1 s.

Despite the performance improvements achieved by multimodal image fusion, both visible light and thermal infrared images still suffer from inherent limitations in dead hen detection tasks. Visible light images are sensitive to low-illumination conditions and show limited discriminative ability between prone live hens and dead hens, while thermal infrared images often produce blurred features for dead hens. More importantly, conventional convolutional neural networks mainly extract static frame-based image features. Whether using single modality or multimodal images, these methods rely on instantaneous visual information and fail to explicitly model temporal dynamics. In caged environments, combining the temporal and spatial dimensions of chicken features for representation and analysis provides an opportunity to capture dynamic changes in health status over time, enabling more accurate health state assessments in a higher dimensional feature space.

Graph neural networks have attracted increasing attention due to their ability to model non-Euclidean structured data, particularly in behavior and posture analysis [18]. Spatial-Temporal Graph Convolutional Networks integrate temporal sequences with graph-structured spatial representations and are capable of jointly modeling spatial and temporal dependencies. In livestock research, applications of graph neural networks are still limited and mainly focus on pigs and cattle. Gan et al. (2022) [19] applied a Spatial-Temporal Graph Convolutional Network to analyze the social behaviors of pre-weaned piglets based on skeletal keypoints obtained from pose estimation. Li et al. (2022) [20] proposed a lameness detection method for dairy cows by combining convolutional networks based on RGB images and optical flow with a Spatial-Temporal Graph Convolutional Network based on skeleton data, achieving an accuracy of 97.20%. Parmiggiani et al. (2023) [21] addressed long-term pig tracking in dense and crowded environments by constructing graph structures from detection and tracking results and applying graph neural networks for identity association, achieving improved tracking accuracy compared with DeepSORT.

To address the limitations of static dead hen detection methods under caged conditions, including crowding, occlusion, and insufficient feature representation, this study proposes a dead hen detection method based on a Spatial-Temporal Graph Convolutional Network using a fundamentally different data representation and recognition strategy. Chicken states are represented using spatial-temporal graph structures. During data preprocessing, cage images are processed to remove cage bars, and multimodal fusion images are generated from visible light and thermal infrared images. A pose tracking algorithm is then applied to estimate skeletal keypoints for each chicken in the cage, enabling construction of individual posture representations. Based on temporal posture sequences, spatial-temporal graph-structured data are constructed for each chicken, where skeletal keypoints are defined as graph nodes, connections between keypoints as spatial edges, and corresponding keypoints across consecutive frames as temporal edges. These spatial-temporal graphs are used as input to a Spatial-Temporal Graph Convolutional Network, which replaces conventional convolutional neural networks to achieve dead hen identification based on dynamic temporal information. The proposed model consists of temporal sequence convolution for extracting temporal features and spatial graph convolution for extracting spatial features.

## 2. Materials and Methods

### 2.1. Data Processing and Construction

#### 2.1.1. Data Acquisition and Preprocessing

The experimental data used in this study were collected in a commercial caged laying hen house located in Chaozhou City, Guangdong Province, China, from 16 April to 29 April 2024. The poultry house adopted an H type cage system with four tiers, and each cage housed 6 to 8 hens. The experimental subjects were yellow feather laying hens aged approximately 47 to 49 weeks. The light intensity in the chicken coops ranges from 10 to 20 lux. Each coop houses approximately 30,000 chickens, and the frequency of dead chickens is about 8 or so per day at most.

**Figure 1:**
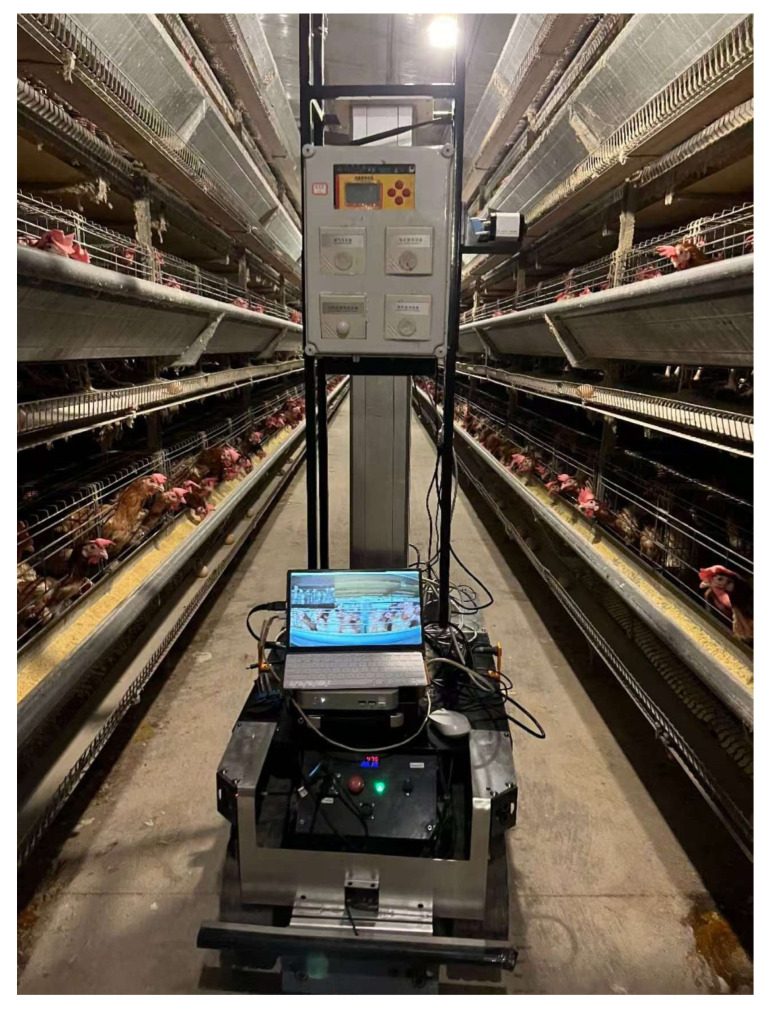
Data collection platform and on-site diagram.

During data acquisition, an inspection robot performed automatic patrols at a constant speed of 0.2 m/s. Video data were captured using a visible light camera (SONY IMX291, Tokyo, Japan) and a thermal infrared camera (IRay, AT61P, Shanghai, China) mounted on a support bracket. To improve the robustness of the detection model, data were collected under varying conditions, including different shooting distances, camera heights, and lighting environments. The data acquisition platform and on-site experimental setup are shown in Figure 1.

During the data acquisition period, approximately 12 h of visible light video and 12 h of thermal infrared video were collected. The resolution of the visible light videos was 1920×1080, while the resolution of the thermal infrared videos was 640×512. Both video streams were recorded at a frame rate of 25 FPS. In total, approximately 30,000 hens were captured in the videos, including 235 dead hens. The health status of each hen was confirmed by professional farm staff. During data acquisition, the time periods in which dead hens appeared in the videos were recorded based on cage locations, facilitating subsequent annotation.

To avoid data leakage, the training, validation, and test sets were constructed using data collected on different days. To eliminate the influence of cage bars on visual features, a cage bar removal algorithm proposed in a previous study [22] was applied to remove cage structures from the images, resulting in complete hen silhouettes. The ground truth labels for “dead” and “healthy” hens were assigned through a video-level manual annotation process based on behavioral observation. Specifically, a hen was labeled as “dead” if it exhibited continuous immobility throughout the video clip, combined with specific abnormal postures such as lying prone with no visible respiratory motion. Conversely, a hen was labeled as “healthy” if it displayed normal physiological behaviors, such as standing, walking, feeding, or slight body movements during rest.

To ensure accurate pixel-level correspondence between the visible light and thermal infrared images, a registration method based on camera calibration parameters was implemented before inputting the data into the fusion model. The specific registration process consists of three key steps: camera calibration, distortion correction, and affine transformation. Firstly, we utilized a custom-made calibration board (180×135 mm) featuring a 12 × 9 checkerboard pattern. To ensure visibility in both modalities, the board was equipped with heating elements to create thermal contrast for the thermal infrared camera. Both cameras were calibrated using the MATLAB Camera Calibration Toolbox (version R2022b, MathWorks, Natick, MA, USA). Then, distortion correction was carried out based on the camera intrinsic parameters obtained through calibration. Since the visible light and thermal infrared cameras have different resolutions and fields of view (FOVs), an affine transformation was required to align the thermal infrared images to the visible coordinate system. A scale factor matrix was calculated based on the pixel sizes and focal lengths of the two cameras. Translational alignment was performed by extracting the centroids of the checkerboard corners from 20 pairs of synchronized calibration images. The median differences between the feature points in the visible light and thermal infrared images were computed to determine the optimal global offset.

Subsequently, the SuperFusion algorithm [23] was used to fuse visible light and thermal infrared data, providing multimodal inputs for subsequent pose estimation and Spatial-Temporal Graph Convolutional Network modeling. The final resolution of the fused images was 640×512.

In this section, the YOLOv7-Pose algorithm was first employed to predict the pose keypoints of hens within the cages. The pose estimation model was trained and evaluated using 3000 static multimodal fused images that were split into training, validation, and test sets with a ratio of 6:2:2. In addition, spatial-temporal graph-structured data were constructed from video sequences, comprising a total of 834 hen video samples. For the Spatial-Temporal Graph Convolutional Network model, hen health status was categorized into two classes, namely healthy and dead, containing 578 and 256 video samples, respectively. These data were also divided into training, validation, and test sets with a ratio of 6:2:2 to ensure model generalization and robustness. The static images used for pose estimation and the video data used for the Spatial-Temporal Graph Convolutional Network were derived from the same original dataset, with static images extracted directly from the video sequences.

#### 2.1.2. Construction of Chicken Spatial Pose Data

**Figure 2:**
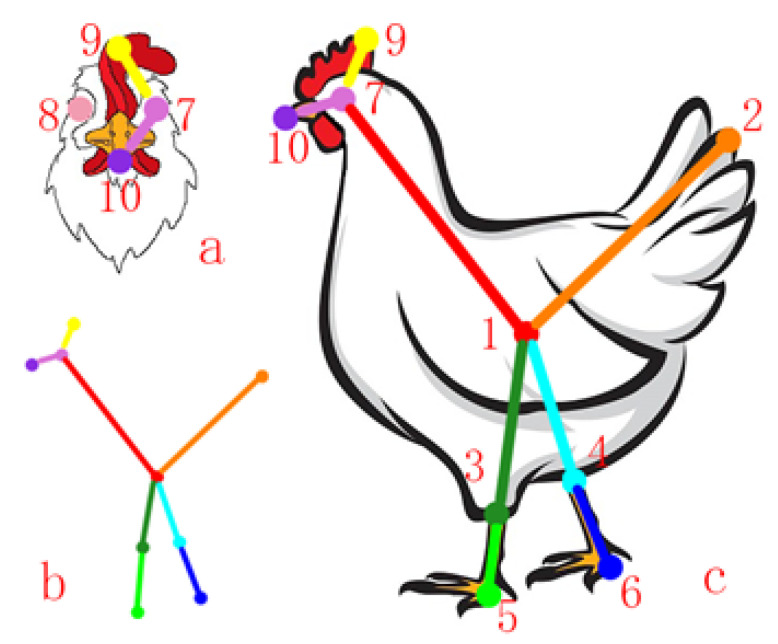
Definition of chicken skeletal keypoints proposed by Fang et al. (2021) [24]. The keypoint indices (1–10) represent Body center, Tail, Left knee, Right knee, Left heel, Right heel, Left eye, Right eye, Comb, and Beak, respectively.

Chicken posture and behavior can directly reflect health status. In this study, the spatial posture of individual hens is represented using skeletal features following the keypoint annotation scheme and connection relationships proposed by Fang et al. (2021) [24]. This scheme defines a total of 10 skeletal keypoints and 11 keypoint connection relationships to accurately describe chicken posture information. The definition of chicken skeletal keypoints is illustrated in Figure 2, where keypoints indexed from 1 to 10 correspond to the body center, tail, left knee, right knee, left heel, right heel, left eye, right eye, comb, and beak, respectively.

Specifically, the body center is defined as the center of the maximum inscribed circle within the contour boundary of the chicken. The tail point is determined as the intersection between the chicken contour and the longest line extending from the body center toward the tail region. The left and right knee points are located at the color boundary between the legs and feathers. The left and right heel points correspond to the joint positions of the phalanges. The left and right eye points are defined as the centers of the maximum inscribed circles within the eye contours. The comb point is located at the terminal point of the boundary between the comb and feather regions, while the beak point is annotated at the foremost vertex of the beak.

The connections between skeletal keypoints describe the relative spatial relationships between pairs of keypoints and enable effective representation of chicken skeletal features. The connection relationships among all skeletal keypoints are listed in Table 1. In this skeletal structure, the body center is connected to the tail, eyes, and feet to form the main skeletal framework. Subsequently, the eyes are connected to the beak and comb to construct the head skeletal structure.

In this study, Labelme (version 5.2.1, Wada Kentaro, Tokyo, Japan) software was used to annotate chicken skeletal keypoints, and the bounding boxes of chicken contours were also annotated. Based on the bounding boxes, chickens were categorized into two classes, namely “healthy” and “dead”. This categorical information was used for preliminary classification of chicken health status at the static image level during the pose tracking stage. In caged environments, due to occlusion caused by feeders and mutual obstruction among chickens, only visible keypoints were annotated. Keypoints that were invisible or severely occluded were not annotated.

#### 2.1.3. Construction of Spatial-Temporal Graph-Structured Data

Graph structures are a representation form for non-Euclidean data and consist of a node set and an edge set that jointly define the topology. Nodes represent entity features, whereas edges describe interactions between entities. On this basis, spatial-temporal graph structures introduce a time dimension. By dynamically updating node attributes over time, they can capture both spatial topological relationships and the temporally evolving behavior patterns of targets, enabling effective modeling of continuity in biological behaviors. This property makes spatial-temporal graphs a suitable mathematical representation for analyzing sequential behaviors and provides theoretical support for extracting deeper behavioral semantics from video streams.

In this study, behavioral videos were collected using an inspection robot, and dynamic chicken skeletal features were extracted from the videos to represent chicken behaviors over time, thereby constructing spatial-temporal graph-structured data. First, following the construction procedure of chicken spatial pose data, the contour and keypoint features of each chicken in the cage were extracted. Skeletal features of each chicken were tracked across consecutive frames, and the tracked sequences were saved as individual chicken videos. After this processing, each original video clip containing multiple chickens was decoupled into multiple single chicken video clips. Then, a pose extraction script together with a pre-trained pose estimation model was used to automatically extract chicken skeletal features for each frame in the video clips.

In the implementation of the chicken spatial-temporal graph structure, skeletal keypoints are defined as the nodes of the graph, while edges are defined according to the skeletal keypoint connection relationships. The spatial dimension of the graph structure is defined as Equation (1).

(1) G=(V,E),

where *V* denotes the set of nodes and *E* denotes the set of edges.

A node feature matrix is defined as $X^{v}\in R^{n\times d}$, where the feature vector of a node *v* is denoted as $x_{v}\in R^{d}$. Here, *n* represents the number of nodes and n=10. Node features are defined using the keypoint coordinates *x*, *y*, and the confidence score *c* obtained from pose estimation, and are expressed as Equation (2).

(2) $$x_{v}=\left(x,y,c\right).$$

According to the connection relationships among chicken skeletal keypoints, spatial edges in the graph structure are defined as undirected edges. The edge feature set is defined as $X^{e}\in R^{m\times d}$, where *m* denotes the number of edges and m=11.

**Figure 3:**
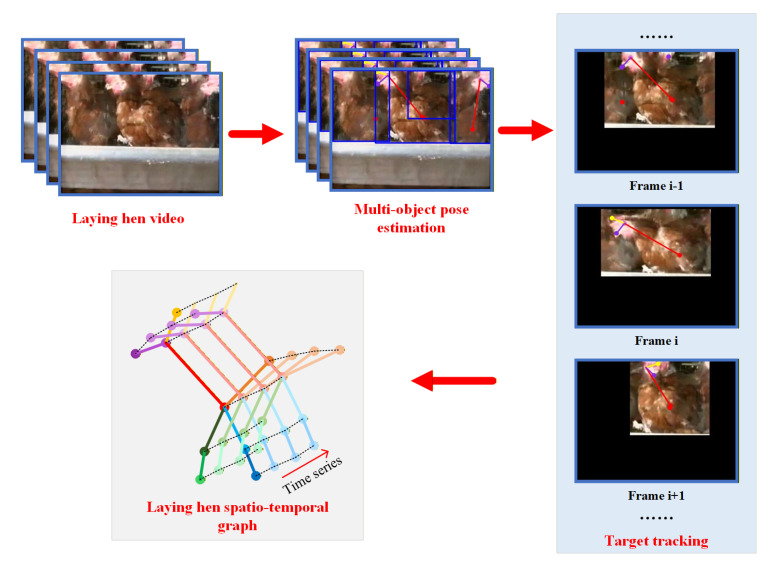
Construction process of chicken spatial-temporal graph-structured data.

Temporal edges in the spatial-temporal graph structure connect the same node *v* across different time steps. These temporal edges explicitly link identical joints in adjacent frames, forming adjacency relationships along the temporal dimension. The construction process of chicken spatial-temporal graph-structured data is illustrated in Figure 3.

### 2.2. Overall Framework

**Figure 4:**
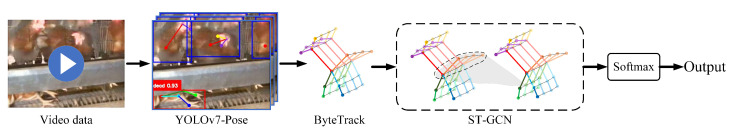
Overall architecture.

The proposed dead hen identification model based on a Spatial-Temporal Graph Convolutional Network integrates the spatial posture features of chickens with temporal variations to achieve dead hen recognition in caged environments at a higher feature dimension. First, the YOLOv7-Pose algorithm is employed to detect chicken pose keypoints and locations, and the ByteTrack target tracking algorithm is used to track chicken postures within cages. This process enables acquisition of sequential posture data for each individual chicken over a predefined time interval. Subsequently, the decoupled spatial-temporal graph-structured data of individual chickens are fed into the Spatial-Temporal Graph Convolutional Network for prediction, thereby achieving dead hen identification based on dynamic spatial-temporal information. The overall framework of the proposed method is illustrated in Figure 4.

### 2.3. Pose Tracking Method for Caged Chickens

YOLO-Pose is a pose estimation method built on the YOLO object detection framework. It is a heatmap-free keypoint detection approach for 2D multi-object pose estimation. In a single forward pass, the model predicts both bounding boxes and the corresponding pose keypoints. Unlike conventional pose estimation pipelines, YOLO-Pose follows the standard post-processing procedure of object detection and avoids complex bottom up preprocessing steps. The model is trained in an end-to-end manner without relying on separate post-processing.

**Figure 5:**
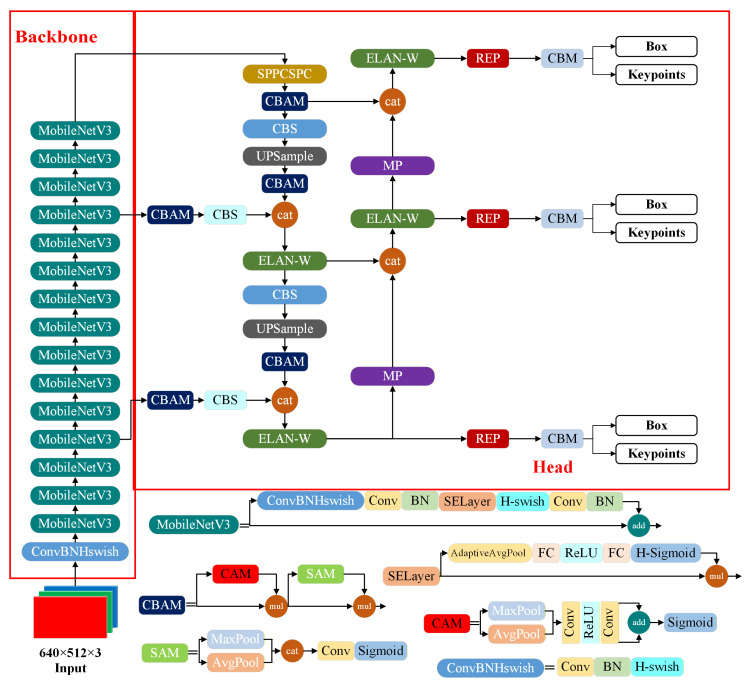
Network architecture of the YOLOv7-Pose pose estimation model.

In our previous work, an improved YOLOv7 network was proposed [13] that achieved strong performance under mutual occlusion among chickens and low illumination, while also providing advantages in inference speed. Therefore, this study continues to adopt this network as the backbone for chicken pose estimation, with task-specific modifications for keypoint prediction. Specifically, the YOLOv7-Pose model used in this study shares the same backbone network and feature aggregation network as the improved YOLOv7 network. The main difference is that YOLOv7-Pose adds a keypoint regression branch on top of the original detection head. As a result, the model outputs not only bounding boxes, class labels, and confidence scores, but also pose-related keypoint information, including the coordinates and confidence score of each keypoint. The network architecture of the improved YOLOv7-Pose used in this study is shown in Figure 5.

Compared with other tracking algorithms, ByteTrack demonstrates superior performance under occlusion conditions by considering a larger number of low-confidence detection boxes, which effectively reduces missed detections [25]. When a target chicken is occluded, the tracking target can be associated with low-confidence detections. Once the occluded target reappears, it can be re associated with high-confidence detections. This mechanism makes ByteTrack particularly suitable for caged environments. Therefore, ByteTrack is selected as the target tracking method in this study.

In summary, by combining the YOLOv7-Pose pose estimation algorithm with the ByteTrack target tracking algorithm, accurate and efficient pose tracking of individual chickens within cages can be achieved. This provides reliable posture data for the subsequent dead hen identification model.

### 2.4. Spatial-Temporal Graph Convolutional Network Model

Regular grid data such as images and videos can be processed using conventional convolutional neural networks, in which data features are extracted through sliding window operations with local receptive fields. By using weight sharing and spatial translation invariance, local features can be effectively learned. However, many types of data in the real world are naturally represented in graph structures, including traffic networks, biological molecular structures, skeletal structures, and higher-dimensional spatial-temporal data. These data exhibit non-Euclidean characteristics, where connections between certain nodes are irregular or even dynamically changing. Conventional convolution operations rely on regular neighborhood structures and therefore cannot be directly applied to such data.

**Figure 6:**
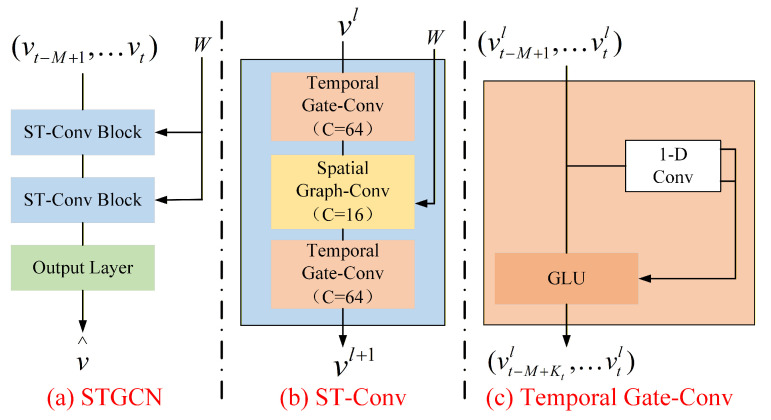
Architecture of the Spatial-Temporal Graph Convolutional Network. (**a**) STGCN; (**b**) ST-Conv; (**c**) Temporal Gate-Conv.

The dead hen identification model investigated in this study relies on spatial-temporal information and therefore cannot be constructed using conventional convolution operations on regular grids. Instead, a Spatial-Temporal Graph Convolutional Network is adopted. This graph convolutional architecture is designed for spatial-temporal sequence data and can effectively model temporal dynamics and spatial dependencies in chicken behaviors. The overall architecture of the Spatial-Temporal Graph Convolutional Network is shown in Figure 6a. It consists of two spatial-temporal convolution blocks and a fully connected output layer. Each spatial-temporal convolution block includes two gated temporal convolution layers and one spatial graph convolution layer.

As shown in Figure 6b, residual connections and a bottleneck strategy are adopted within each spatial-temporal convolution block. The input sequence $v_{t-M+1},\ldots ,v_{t}$ is processed by the spatial-temporal convolution blocks in a unified manner to extract spatial and temporal dependencies. The outputs of the spatial-temporal convolution blocks are then aggregated through a fully connected output layer to generate the final prediction.

Spatial graph convolution is directly applied to the graph structure of a single frame to extract spatial dependencies within the chicken skeleton graph at that frame, given a graph G=(V,E,W), where *V* denotes the node set corresponding to chicken skeletal keypoints, *E* denotes the edge set, and $W\in R^{n\times n}$ represents the weighted adjacency matrix.

To reduce the computational cost of the graph convolution kernel Θ, the spatial-temporal graph convolutional network adopts two approximation strategies, namely Chebyshev polynomial approximation and first-order approximation. The Chebyshev polynomial approximation is suitable for graph structures of moderate scale, whereas first-order approximation is more appropriate for large-scale graph structures with thousands of nodes. Since the graph structure in this study is relatively small, the Chebyshev polynomial approximation is adopted. Under this approximation, the graph convolution can be defined as Equation (3).

(3) $$\Theta {*}_{G}x=\Theta \left(L\right)x\approx \sum_{k=0}^{K-1}\theta_{k}T_{k}\left(\tilde{L}\right)x,$$

where $T_{k}\left(\tilde{L}\right)\in R^{n\times n}$ denotes the *k*-th order Chebyshev polynomial evaluated at the scaled Laplacian $\tilde{L}=\frac{2L}{\lambda_{\max}}-I_{n}$. The Chebyshev polynomial approximation enables recursive computation of *K*-hop localized graph convolutions. Here, $\theta_{k}$ denotes the polynomial coefficients, and *K* represents the order of the Chebyshev polynomial.

The gated temporal convolution operates on each individual node and processes the temporal sequence associated with that node. The Spatial-Temporal Graph Convolutional Network employs a full convolutional structure along the temporal dimension to capture the temporal dynamics of chicken behaviors, as illustrated in Figure 6c. Specifically, the temporal convolution layer consists of a one dimensional convolution with kernel width $K_{t}$, followed by a gated linear unit that serves as the nonlinear activation function.

For each node in the graph G, temporal convolution explores the $K_{t}$ neighboring elements of the input sequence without padding, which results in a reduction of the sequence length by $K_{t}-1$ after each temporal convolution. Accordingly, the temporal convolution input for each node can be viewed as a sequence of length *M* with $C_{i}$ channels, denoted as Equation (4).

(4) $$Y\in R^{M\times C_{i}}.$$

The convolution kernel is defined as Equation (5).

(5) $$\Gamma \in R^{K_{t}\times C_{i}\times 2C_{o}},$$

which maps the input Y to a single output element, as Equation (6).

(6) $$\left[P,Q\right]\in R^{\left(M-K_{t}+1\right)\times 2C_{o}}.$$

The gated temporal convolution can then be expressed as Equation (7).

(7) $$\Gamma {*}_{T}Y=P⊙\sigma (Q)\in R^{\left(M-K_{t}+1\right)\times C_{o}},$$

where P and Q denote the gate inputs of the gated linear unit, ⊙ represents element wise multiplication, and σ(·) denotes the Sigmoid function.

Spatial-temporal convolution blocks are designed to fuse spatial and temporal features by jointly processing graph-structured temporal sequences. The intermediate spatial graph convolution layer is placed between two temporal convolution layers, enabling propagation of spatial states through temporal convolutions. In addition, the sandwich structure of the spatial-temporal convolution block facilitates effective use of the bottleneck strategy by performing downsampling and upsampling on the channel dimension *C*, thereby achieving scale compression and feature squeezing. Furthermore, layer normalization is applied within each spatial-temporal convolution block to prevent overfitting.

### 2.5. Evaluation Metrics

In the object detection branch of YOLOv7-Pose, three evaluation metrics were used, namely precision, recall, and mAP. To evaluate keypoint detection performance, we adopted *P*, *R*, and AP to quantify the effectiveness of chicken pose estimation. These keypoint evaluation metrics were computed based on the Object Keypoint Similarity (OKS), which is analogous to IoU in object detection. OKS measures the similarity between predicted keypoints and ground-truth keypoints by considering both localization error and the importance weighting of keypoints [26]. The definitions of *P*, *R*, AP, and OKS are given in Equations (8)–(11).

(8) $$P=\frac{TP}{TP+FP}$$

(9) $$R=\frac{TP}{TP+FN}$$

where TP denotes the number of true positive samples correctly predicted, FP denotes the number of false positive samples, and FN denotes the number of false negative samples. When the IoU between the predicted bounding box and the ground-truth bounding box is greater than the threshold of 0.5, the prediction is considered correct and counted as TP; otherwise, it is counted as FP.

(10) $$AP_{\mathrm{point}=11}=\frac{1}{11}\sum_{\tilde{k}\in \left\{0,0.1,\ldots ,1\right\}}\max_{\hat{k}\geq \tilde{k}}P\left(\hat{k}\right),$$

where $P(\hat{k})$ denotes the precision at recall level $\hat{k}$ and $\max_{\hat{k}\geq \tilde{k}}P\left(\hat{k}\right)$ represents the maximum precision value selected over all recall levels greater than or equal to $\tilde{k}$.

(11) $$\mathrm{OKS}=\frac{\sum_{i}\exp\;\left(-\frac{d_{i}^{2}}{2s^{2}k_{i}^{2}}\right)\;\delta \left(v_{i}\right)}{\sum_{i}\delta \left(v_{i}\right)},$$

where $d_{i}$ denotes the Euclidean distance between the predicted location and the ground-truth location of the *i*-th keypoint, *s* is the square root of the area of the target detection bounding box and serves as a scale normalization factor, $k_{i}$ is the normalization constant of the *i*-th keypoint, and $v_{i}$ denotes the visibility of the *i*-th keypoint. Specifically, $v_{i}=0$ indicates that the keypoint is not labeled, $v_{i}=1$ indicates that the keypoint is labeled but not visible, and $v_{i}=2$ indicates that the keypoint is labeled and visible. The function $\delta (v_{i})$ is an indicator function.

In this study, the average precision and average recall for pose estimation are computed under an OKS threshold of 0.5. The evaluation criteria used for pose estimation are consistent with those used in object detection tasks. A prediction is considered a true positive when the OKS is greater than or equal to the predefined threshold; otherwise, it is regarded as a false positive.

(12) $$\mathrm{Accuracy}=\frac{TP+TN}{TP+TN+FP+FN}$$

where TP, TN, FP, and FN denote the numbers of true positives, true negatives, false positives, and false negatives, respectively. Classification accuracy reflects the proportion of samples that are correctly predicted by the model among all samples.

## 3. Results and Discussion

### 3.1. Experimental Settings

The experimental settings of this study are summarized in Table 2. All proposed dead hen detection models were trained on a Windows-based workstation (Windows 11 Pro) equipped with an NVIDIA RTX 3090 GPU and an Intel Core i7-12700F CPU. All experiments were conducted in a Python 3.7.10 environment, and model implementation and training were performed using PyTorch 1.7.1. In addition, CUDA 11.1 and cuDNN 8.0.4 were used to accelerate computations during training, leveraging GPU parallelism to improve training efficiency and overall performance.

The hyperparameter configuration of the pose estimation model for caged chickens is shown in Table 3.

The chicken spatial-temporal data in this study follow the Kinetics-Skeleton dataset format, in which the video length ranges from 30 to 60 frames. Data augmentation methods including random frame selection and random movement were applied. The hyperparameters of the Spatial-Temporal Graph Convolutional Network are presented in Table 4. The temporal window size was set to 200. If the input sequence contained fewer than 200 frames, data augmentation was used for padding. In addition, the initial learning rate was 0.001, the batch size was 16, and the number of iterations was 1000.

### 3.2. Architecture and Analysis of the Pose Estimation Model

Both quantitative and qualitative analyses were conducted on the proposed pose estimation results for caged chickens. The improved YOLOv7-Pose model performs joint prediction of chicken categories and pose keypoints, including an object detection branch and a keypoint detection branch. In object detection, the model achieved a precision of 97.9% and a recall of 97.4% for the dead class, and a precision of 95.8% and a recall of 95.2% for the healthy class. The improved backbone network provides favorable inference efficiency while remaining compatible with the keypoint detection task and maintaining strong overall performance.

For keypoint detection, under an OKS threshold of 0.5, the model achieved an average precision of 92.8%, an average recall of 92.3%, and an average precision of 92.6%. The proposed pose estimation model is able to detect most of the visible skeletal keypoints. However, due to mutual occlusion among chickens and occlusion caused by feeders, it is difficult to detect all skeletal keypoints completely in crowded caged environments. The test results of the pose estimation model are reported in Table 5. The results indicate that keypoint detection performance for dead hens is lower than that for healthy hens. This is mainly because dead hens often present irregular postures, leading to skeletal features that differ substantially from those of healthy hens, and they are more likely to be affected by occlusion.

**Figure 7:**
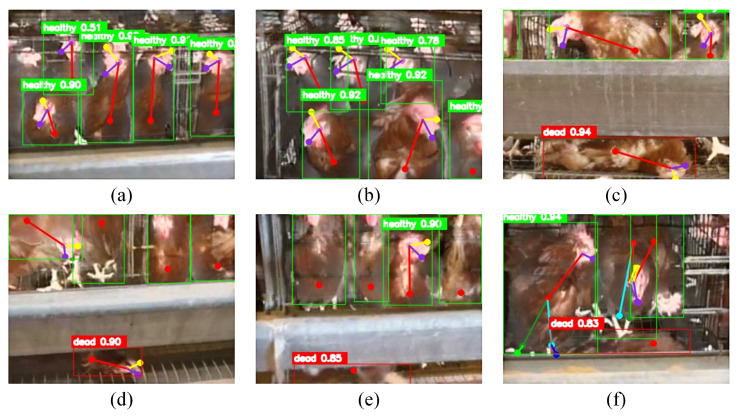
Pose estimation model prediction results. (**a**,**b**) no dead chickens; (**c**–**f**) dead chickens present.

The qualitative test results of the pose estimation model for caged chickens are shown in Figure 7, which includes the prediction results for six chicken images. In practical poultry house environments, due to occlusion caused by feeders, skeletal keypoints on the lower body of chickens are often not visible. In addition, because the metal mesh supporting the chickens inside the cage is inclined, dead hens tend to appear near the bottom of the feeder. Although placing the camera in a top down view can provide richer skeletal keypoint information, this viewpoint also tends to occlude dead hens located at the bottom of the feeder. During the experiments, it was observed that the upper body skeleton alone is sufficient to represent the health status of chickens. The visible keypoints mainly include the body center point, beak point, comb point, and the left eye or right eye point. Clear differences were also observed between healthy and dead chickens in the posture features represented by these visible keypoints.

The qualitative results indicate that the proposed pose estimation model achieves reliable detection performance when the chicken contour and posture are visible, and it can still accurately predict both chicken categories and keypoints in crowded caged environments. The object detection branch achieved the expected performance. Compared with previous studies based on single-modality visible-light images, the proposed model provides improved detection results, mainly because multimodal fusion images offer richer feature information.

For keypoint detection, the model performs better on healthy chickens than on dead hens. As shown in Figure 7a,b, the visible keypoints of healthy chickens can be accurately detected. This is mainly because after death, the body contour of a chicken often becomes irregular and rigid. In addition, dead hens are more likely to be occluded by other chickens and by feeders, and the model may only detect the body center point in some cases, as shown in Figure 7e,f. Therefore, keypoint detection for dead hens is substantially more challenging than for healthy chickens. Nevertheless, for most dead hens, the model can still detect the visible skeletal keypoints reasonably well, as shown in Figure 7c,d. Moreover, in crowded cages, the skeletal keypoints of different individuals may be spatially close, which can occasionally lead to incorrect inter-individual keypoint connections. For example, in Figure 7f, the body center point of the chicken in the back is mistakenly connected to the left eye point of the chicken in the front. Overall, although partial misdetections may occur under occlusion, the proposed pose estimation model can accurately predict the category and visible keypoints for most dead and healthy chickens.

Pose estimation serves two purposes in this study. First, it provides skeletal features that form the basis of spatial-temporal data for the subsequent spatial-temporal prediction model. Second, it provides an initial static classification of caged chicken categories, which is further refined by combining spatial-temporal prediction results. Based on the spatial-temporal graph structure obtained after ByteTrack tracking, the proposed pose estimation model can provide accurate keypoint coordinates under different imaging conditions and chicken postures. This enables construction of an appropriate spatial-temporal graph structure across the full time series and supports accurate representation of dynamic spatial-temporal behaviors. Although the pose estimation model only provides a two dimensional and static assessment of chicken health status, its outputs remain important for the final decision.

### 3.3. Results and Analysis of the Spatial-Temporal Graph Convolutional Network Model

The test results are summarized in Table 6. The proposed Spatial-Temporal Graph Convolutional Network achieved an overall classification accuracy of 99.0%, while the classification accuracy for the dead hen class reached 98.9%. Benefiting from the strong capability of the spatial-temporal graph convolutional network to extract spatial and temporal features, dead hens in caged environments can still be accurately identified even under severe occlusion conditions. In contrast, object detection models are prone to false detections or missed detections when target features are partially missing due to occlusion. The proposed dead hen identification model based on dynamic spatial-temporal information effectively alleviates the negative impact of single-frame occlusion by leveraging temporal dynamics and spatial relations across consecutive frames.

In our previous studies, we conducted the detection of caged dead chickens based on single-modality visible-light images and multimodal fused images, respectively. The experimental results show that the detection precision of dead chickens using single-modal visible images is 95.7%, while that using multimodal images is 97.6%. Whether it is a single-modal or multimodal image, they are all based on static image data and traditional convolutional neural networks. When dealing with occluded situations, there will still be a certain degree of false detection and missed detection [27]. The spatiotemporal model proposed in this paper can effectively solve the problems of occlusion. The achieved accuracy of 99.0% is superior to that of single-modal and multimodal static images.

During dead hen identification with the Spatial-Temporal Graph Convolutional Network, occlusion may cause some dead hens to have only one detected keypoint in pose estimation, or even no detected keypoints. However, the impact of such missing keypoints is limited over the full temporal sequence of the spatial-temporal graph. As the inspection robot moves, subsequent frames may capture previously occluded body parts more clearly. Moreover, the dynamic variation patterns in spatial-temporal graph data may serve as evidence for dead hen identification. Therefore, by elevating the representation to the dynamic spatial-temporal dimension, the model tolerance to missing features is improved, enabling relatively accurate dead hen identification even when partial features are absent.

Because the test images used for pose estimation were extracted from the test videos used for the Spatial-Temporal Graph Convolutional Network, the two test sets originate from the same dataset. This section provides a joint analysis of the test results of both models and discusses the causes of incorrect predictions. The pose estimation test set contains 600 images that were extracted from 166 test videos. These videos are identical to the test videos used by the Spatial-Temporal Graph Convolutional Network. In the object detection task, each sample corresponds to a single image, whereas the Spatial-Temporal Graph Convolutional Network is trained and evaluated on spatial-temporal graph structures, where each sample corresponds to an entire spatial-temporal graph.

**Figure 8:**
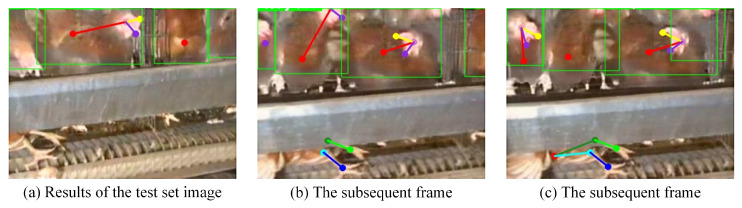
Examples of incorrect predictions.

In pose estimation, the number of samples with incorrect category predictions is 56, originating from 26 videos. In contrast, for the Spatial-Temporal Graph Convolutional Network, there are only three incorrect predictions among these 26 videos. These results indicate that the Spatial-Temporal Graph Convolutional Network substantially improves the accuracy of chicken health status detection by capturing spatial-temporal dependencies in dynamic behaviors. This improvement is mainly attributed to the ability of the model to capture temporal variations through temporal convolution, aggregate spatial relations through graph convolution, and suppress noisy frames through the gating mechanism, thereby enhancing dynamic spatial-temporal detection performance for dead hens in caged environments. Figure 8 presents misprediction cases from the pose estimation model and also shows the detection results on other frames from the corresponding videos.

In Figure 8a, a dead hen is located at the bottom of the feeder, with only two legs visible. Due to the lack of distinctive features, the pose estimation model fails to correctly identify this dead hen. To further analyze the spatial-temporal graph constructed from this video, subsequent frames of the same video were also input into the pose estimation model for detection. The corresponding results are shown in Figure 8b,c. In these frames, the pose estimation model is only able to detect partial keypoints of the dead hen and fails to fully localize its contour or assign a correct category.

However, in the prediction of the Spatial-Temporal Graph Convolutional Network, despite partial loss of contextual information in the spatial-temporal graph, the model is still able to accurately identify the dead hen based on spatial-temporal dependency relationships. This demonstrates that the Spatial-Temporal Graph Convolutional Network can effectively compensate for recognition difficulties caused by feature loss or occlusion in pose estimation and can produce more accurate decisions by leveraging dynamic spatial-temporal information.

To verify the effectiveness of the proposed spatial-temporal approach, we compared the final classification results with the performance of YOLOv7-Pose, which relies solely on single-frame spatial features. The spatial-only baseline achieved an precision of 97.9%. The analysis of the results revealed that the main limitation of the single-frame method was the inability to distinguish between resting hens and dead hens, as their geometric postures are highly similar in static images. Additionally, temporary occlusion in crowded cages often led to missed detections in the spatial model. In contrast, by integrating the ST-GCN, the proposed method significantly improved the accuracy to 99.0%. The temporal dynamics captured by the ST-GCN enabled the system to differentiate the absolute immobility of dead hens from the subtle physiological movements of resting hens, while also mitigating the impact of occlusion through sequence-level analysis.

Since the proposed method operates as a cascaded framework, the robustness of the final classification depends on its ability to tolerate errors from upstream tasks. Although occlusion or lighting changes may cause the upstream YOLOv7-Pose model to generate jittery keypoints or missed detections in individual frames, the proposed ST-GCN demonstrates strong robustness to such noise. This robustness is primarily attributed to the temporal aggregation mechanism of the ST-GCN. The model does not rely on a single frame but extracts features from a continuous spatial-temporal sequence. Therefore, sporadic missing keypoints or local tracking deviations are treated as high-frequency noise and are effectively filtered out by the spatial-temporal convolution operations. The model focuses on global behavioral patterns rather than instantaneous skeletal states. As evidenced by the comparative results, although the upstream pose estimation stage produced 56 misclassifications due to visual ambiguity, the integrated ST-GCN system successfully corrected these errors, achieving a final accuracy of 99.0%. This confirms that the temporal context significantly mitigates the impact of upstream detection failures. The proposed method takes approximately 126 ms to process each image, with a frame rate of about 8 FPS. Currently, this method cannot achieve real-time detection on the devices used in the experiments. Therefore, improving the efficiency of the method is also a major task for the future.

In practical deployment, high-accuracy detection is only the first step. A complete on-farm system should convert frame-level detections and temporal decisions into actionable reporting for staff, such as an automatic alert when a cage shows sustained immobility consistent with a dead hen, together with a short natural language report that includes cage identifier, timestamp, confidence score, and a brief summary of recent events. Large language model-based summarization can be considered to generate concise reports from structured event logs [28].

In summary, the proposed dead hen identification model achieves strong overall performance. Moreover, since the recognition accuracy in this study is computed based on multi-frame sequences rather than single-frame predictions, the evaluation results are more representative than static metrics. The proposed method exhibits clear innovation in both data representation and modeling strategy, and provides a valuable reference for behavior recognition in livestock and poultry.

## 4. Conclusions

This study addresses the challenges of occlusion and posture ambiguity in dead hen detection within caged poultry houses by proposing an identification method that integrates multimodal pose estimation with a Spatial-Temporal Graph Convolutional Network. Unlike conventional static object detection approaches, the proposed method extracts and tracks chicken skeletal keypoints from fused images using an improved YOLOv7-Pose model and the ByteTrack algorithm, and constructs spatial-temporal graph structured data that represent continuous behavioral patterns of individual chickens. This data representation effectively transforms chicken health status from isolated visual features into topological features with temporal dependencies.

Experimental results demonstrate the superiority of the spatial-temporal graph convolutional model in dead hen identification. The proposed ST-GCN model achieved an average accuracy of 99.0% on the test set. Compared with detection methods that rely solely on single-frame images, the proposed model aggregates dynamic features across temporal sequences, effectively compensating for spatial feature loss caused by feeder occlusion or insufficient illumination. Even when the pose estimation model detects only a subset of keypoints, the ST-GCN can still make accurate decisions by exploiting spatial-temporal contextual information, resulting in robust detection performance under complex environmental conditions.

By leveraging the differences in spatial-temporal behavioral patterns between dead and live chickens, the proposed method exhibits high tolerance and stability when handling samples with inconspicuous visual features caused by crowding or prone postures. Overall, this study introduces innovations in both data representation and modeling dimensions. The proposed approach not only overcomes the limitations of static detection methods but also provides a theoretical foundation and practical reference for future fine-grained livestock and poultry behavior analysis and health monitoring based on non-Euclidean data.

## Figures and Tables

**Table 1:** Skeleton keypoint connection relationships.

No.	Keypoint	Keypoint Connection Relationships
1	Body center	(Body center, Tail), (Body center, Left knee), (Body center, Right knee),
		(Body center, Left eye), (Body center, Right eye)
2	Tail	–
3	Left knee	(Left knee, Left heel)
4	Right knee	(Right knee, Right heel)
5	Left heel	–
6	Right heel	–
7	Left eye	(Left eye, Comb), (Left eye, Beak)
8	Right eye	(Right eye, Comb), (Right eye, Beak)
9	Comb	–
10	Beak	–

**Table 2:** Experimental parameter settings.

Configuration	Parameter
CPU	Intel Core i7-12700F
GPU	NVIDIA RTX 3090
Operating System	Windows 11
GPU Computing Platform	CUDA 11.1
GPU Acceleration Library	cuDNN 8.0.4
Deep Learning Framework	PyTorch 1.7.1

**Table 3:** Hyperparameters of the pose estimation model.

Hyperparameter	Value
Optimizer	Adam
Learning rate	0.01
Momentum	0.937
Weight decay	0.0005
Batch size	16
Iterations	300

**Table 4:** Hyperparameters of the STGCN model.

Hyperparameter	Value
Training strategy	spatial
Learning rate	0.001
Temporal window	200
Batch size	16
Iterations	1000
Video length (frames)	30–60

**Table 5:** Test results of the pose estimation model.

Model	Class	Evaluation Metrics (%)			
		P	R	AP@0.5	AP@0.95
Object detection	All	96.9	96.3	94.6	84.1
	Healthy	95.8	95.2	93.9	83.2
	Dead	97.9	97.4	95.3	85.1
Keypoint detection	All	92.8	92.3	82.6	67.0
	Healthy	94.7	94.2	87.0	65.8
	Dead	90.9	90.4	78.2	68.2

**Table 6:** Test results of the STGCN model.

Class	Accuracy (%)
Average	99.0
Healthy	99.0
Dead	98.9

## Data Availability

The data presented in this study are available upon request from the corresponding author.
